# Dynamic Corridor Illusion in Pigeons: Humanlike Pictorial Cue Precedence Over Motion Parallax Cue in Size Perception

**DOI:** 10.1177/2041669520911408

**Published:** 2020-03-24

**Authors:** Yuya Hataji, Hika Kuroshima, Kazuo Fujita

**Affiliations:** Department of Psychology, Graduate School of Letters, Kyoto University

**Keywords:** size constancy, pictorial depth cue, motion parallax, pigeons

## Abstract

Depth information is necessary for perceiving the real size of objects at varying visual distances. To investigate to what extent this size constancy present in another vertebrate class, we addressed the two questions using pigeons: (a) whether pigeons see a corridor illusion based on size constancy and (b) whether pigeons prioritize pictorial cues over motion parallax cues for size constancy, like humans. We trained pigeons to classify target sizes on a corridor. In addition, we presented a dynamic version of corridor illusion in which the target and corridor moved side by side. Target speed was changed to manipulate motion parallax. With the static corridor, pigeons overestimated the target size when it was located higher, indicating that pigeons see a corridor illusion like humans. With the dynamic corridor, the pigeons overestimated the target size depending on target position, as in the static condition, but target speed did not affect their responses, indicating that the pictorial precedence also applies to pigeons. In a follow-up experiment using the same stimulus, we confirmed that humans perceive object size based on pictorial cues. These results suggest that size constancy characteristics are highly similar between pigeons and humans, despite the differences in their phylogeny and neural systems.

Animals with eyes are faced with the issue of how to reconstruct three-dimensional representations from two-dimensional retinal images to function adequately in their environments (e.g., deciding to chase prey). Humans make use of a variety of cues including binocular disparity, motion parallax, and pictorial depth (Howard, 2012). Which depth cues are used differ among species depending on their morphologies and optical environments (e.g., accommodation cue in owls, Wagner & Schaeffel, 1991; image defocus in spiders, Nagata et al., 2012). Comparative studies can help reveal environmental and phylogenetic factors relevant to visual depth perception in the animal kingdom.

Object size perception is affected by visual depth cues (Fineman, 1981). As viewing distance increases, the retinal size of a viewed object decreases. However, we can perceive the true size of the object by using various depth cues. This function, called size constancy, would be beneficial for all sighted animals evaluating the actual size of an object (e.g., potential food, escape hole, or rival). Actually, some nonhuman primates experience a corridor illusion when presented with two-dimensional pictures containing pictorial depth cues (Barbet & Fagot, 2002, 2007; Imura et al., 2008; Imura & Tomonaga, 2009). In the corridor illusion, objects on a corridor background are perceived as bigger the farther away they seem to be. Barbet and Fagot (2002) trained baboons (*Papio papio*) to show a Go response when two pictures of humans on a screen were of different size. In probe trials in which two persons of the same size were presented, they showed more Go responses in the corridor background condition than in no-corridor background condition. Imura et al. (2008) trained chimpanzees (*Pan troglodytes*) to touch the larger of two objects on a screen. They found that the chimpanzees chose the farther one on a corridor background when two objects of identical size were presented and that the accuracy declined when the smaller object was presented on a farther point and the perceived difference in size of two objects diminished. These studies suggest that size constancy using pictorial cues is shared at least in the order primates.

To date, no empirical study has tested the corridor illusion in avian species, although pigeons (*Columba livia*) are known to experience the Ponzo illusion, another size constancy illusion induced by via two converging lines (Fujita et al., 1991, 1993). Several studies have shown that this species is sensitive to various visual depth cues (Cavoto & Cook, 2006; Cook & Katz, 1999; McFadden & Wild, 1986; Reid & Spetch, 1998). For instance, Reid and Spetch (1998) examined pigeons’ discrimination of pictures of 3-D objects from pictures of objects without shading and perspective depth cues. They found that pigeons use both pictorial depth cues for discrimination. Cavoto and Cook (2006) investigated the contribution of relative density, size, and occlusion cues for discriminating the sequential depth ordering of three objects in a virtual environment. Pigeons were rewarded for responding correctly to specific ordering of objects (e.g., a red object was the nearest, and a blue object was farthest). Learning improved as the cues were additively combined, indicating that pigeons use all pictorial depth cues for the ordering discrimination. From these studies, it is hypothesized that birds resemble primates in being sensitive to the corridor illusion. One goal in the present study was to investigate whether pigeons perceive the corridor illusion, known to occur in baboons and chimpanzees.

If pigeons perceive the corridor illusion, it might be possible that different depth cues are used for size constancy depending on the viewing situations. Testing the selectivity of depth cue on size constancy of nonhuman animals could shed light on the similarity of size constancy mechanisms across species. Thus, the second aim of the current study was to test whether cue selectivity in size constancy is similar between humans and pigeons.

In humans, pictorial depth cues appear to predominate in size constancy compared with motion parallax cue (Gregory, 1970; Luo et al., 2007; Wade & Hughes, 1999; Watt & Bradshaw, 2003). Luo et al. (2007) investigated the effects of binocular disparity, motion parallax, and pictorial cues on size constancy in a virtual environment. They found that whereas binocular and pictorial cues contributed to size constancy, motion parallax had no influence on size perception. Watt and Bradshaw (2003) investigated the effects of binocular disparity and motion parallax cues on reaching movement. Although binocular disparity contributed to both transport and grasp components of reaching, motion parallax had no effect on the grasp component. Moreover, some trick arts induce illusory depth through contradicting depth cues (hollow-mask illusion, Gregory, 1970; reverspective art, Wade & Hughes, 1999). In these examples, pictorial cue override the other visual depth cues, further suggesting that motion parallax is secondary to pictorial cues for size constancy in humans.

Previous animal studies using multiple depth cues suggest an additive effect on the computation of depth information (Barbet & Fagot, 2007; Cavoto & Cook, 2006; Reid & Spetch, 1998). For investigating selectivity of depth cues, however, it is advantageous to present a stimulus in which two depth cues signify different depth directions (e.g., a pictorial cue signifying *near* with a motion parallax cue signifying *far*). To this aim, we produced a *dynamic* version of the corridor illusion. A classic corridor illusion stimulus uses a static perspective corridor picture as background, and objects are perceived larger the nearer they are to the vanishing point of the corridor. In our dynamic corridor illusion, by contrast, the background corridor and objects moved sinusoidally side to side so that faster points appeared nearer.

In this study, we first trained pigeons to classify target sizes into two categories (large or small) in static and dynamic corridor conditions, respectively. The target position in the static condition and the target position and speed in the dynamic condition were constant throughout training. We then manipulated target height (pictorial cue) in the static condition to assess the classic corridor illusion in pigeons. We also investigated cue selectivity in size constancy in pigeons by manipulating target height (pictorial cue) and speed (motion parallax) in the dynamic condition. We predicted that, if size constancy occurs in pigeons, they would overestimate target size when the target appears in a higher position (Pictorial-Far [PF]) in the static condition. We also predicted that, if cue selectivity is similar across species, pigeons would overestimate target size when the target is presented in a higher position and moving faster (Pictorial-Far-Motion-Near [PFMN]) and underestimate size when it is presented lower down and moving slower (Pictorial-Near-Motion-Far [PNMF]). Finally, we conducted the same experiment with humans to confirm the precedence of pictorial over motion depth cues in our dynamic corridor illusion, as shown in previous studies (Luo et al., 2007; Watt & Bradshaw, 2003).

## Experiment 1 (Pigeons)

### Methods

#### Subjects

Six male pigeons participated (mean age: 8.5 years, range: 3–15). All had participated in several operant experiments but were naïve to size discrimination tasks. They were individually housed and maintained at 85% to 95% of their free-feeding weights. Water and grit were freely available in the home cage. The experiments were conducted with the approval of the animal experiment committee of the Graduate School of Letters, Kyoto University (No. 16-34).

#### Apparatus

The experiments were conducted in six identical operant chambers (35 × 35 × 35 cm) installed with a 15-in. LCD monitor (Sharp, LL-T1520, or EIZO, FlexScan L357) and touch-sensitive frame (Touch Panel Systems, UniTouch, or Minato Holdings, ARTS-015N-02B). The monitor resolution was 1,024 by 768 pixels. The monitor refresh rate was set to 60 Hz based on the fact that pigeons discriminate motion stimuli at this rate (Dittrich et al., 1998; Qadri et al., 2014), although it is below the critical flicker frequency of pigeons (140 Hz in electroretinogram study, Dodt & Wirth, 1954; 75 Hz in behavioral study, Hendricks, 1966). A grain hopper delivered food rewards through an opening on left-side wall. The experiments were controlled by a personal computer (Mouse Computer, LM-i500SC, or ThirdWave Corporation, Diginnos Series) running MATLAB with the Psychtoolbox extensions (Brainard, 1997).

#### Stimuli

**Figure 1.**
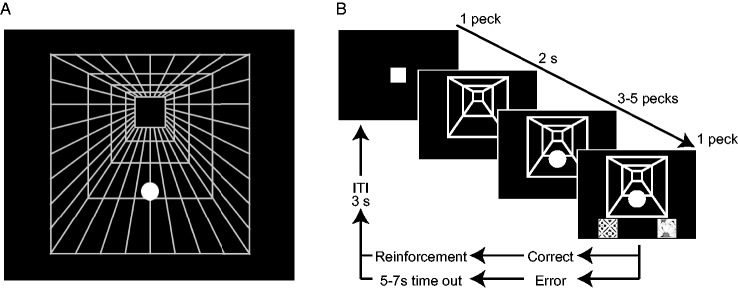
Stimulus and Procedure of Experiment 1. (A) The corridor illusion stimulus used in Experiment 1. One white circle was depicted on a corridor background made of white grid. In the dynamic condition, the circle and background moved horizontally so that the nearer parts moved faster. (B) Schematic illustration of trial sequence in Experiment 1. Note that the relative sizes of stimulus components changed, and the backgrounds was simplified for illustration purpose.

A corridor background was composed of 5 white line squares of different sizes radially (80 to 500 pixels) and 32 radial lines connecting the squares (Figure 1A). The line width was 2 pixels. A single white circle (25, 29, 35, 41, 47, or 55 pixels in diameter) was presented as the target on the corridor. In the static condition, the target was horizontally centered on the corridor. The vertical position of target was different depending on the testing condition (see later). In the dynamic stimulus condition, the corridor and target moved sinusoidally side to side at 0.5 Hz. Movement width of the corridor was 160 and 40 pixels for the largest and smallest squares so that the larger square moved faster (see Supplementary Movie S1). The position and movement width of the target were manipulated according to experimental phases (see later).

#### Procedure

Pigeons were trained on a size classification task (Figure 1B). A trial started with a white square (self-start icon, 50 by 50 pixels) appearing at the center of the display. Pecking the self-start icon immediately replaced it with the background corridor. After 2 s, a target white circle appeared at 135 pixels height from lower edge of the corridor and, in the dynamic condition, moved with 70 pixels width. Three to five pecks at the target produced two square response icons of different textures (50 by 50 pixels) below the corridor. The pigeons had to peck different icons according to the target size (one icon for 25, 29, and 35 pixels and the other icon for 41, 47, 55 pixels). Three pigeons had to peck the left icon if the target size was small, and the other pigeons had to peck the left icon if the target size was large. A single response to the correct icon was reinforced with 2.5 to 6 s access to mixed grain accompanied by a hopper light (primary reinforcement) or hopper light only (secondary reinforcement). A single response to the wrong icon was followed by 5 s time-out, and a correction trial was inserted before the next trial. In a correction trial, the same stimulus appeared, and pecks to the wrong icons were not counted. This procedure was aimed at preventing response biases. The intertrial interval was 3 s. The duration of timeout, access to food, and the probability of secondary reinforcement varied according to the weight and motivation of each subject.

The pigeons were first trained on the largest and smallest targets (25 and 55 pixels) without the corridor background in the static condition. After reaching at least a 90% correct rate, the probability of primary reinforcement was lowered from 100% to 25%, and the number of trials increased from 96 to 360. Then, the dynamic conditions were inserted on the half of trials by increasing the movement width in four steps. The background was gradually faded in in five steps by manipulating the line luminance. Finally, six target sizes were presented in the static and dynamic conditions in one session, in random order.

The pigeons advanced to test sessions after scoring above 85% correct on two consecutive training sessions. Each test session consisted of 360 trials of which 72 were probe trials. Half of the probe trials were the static condition in which the target appeared at a higher (PF) or lower position (PN) than in the training condition (±20 pixels). The other half of the test trials presented dynamic stimuli in which the target appeared at a higher (PF) or lower (PN) position (±20 pixels) and moved slower (MF) or faster (MN, ±8 pixels width). Thus, there were four probe conditions of dynamic stimuli: Pictorial-Far-Motion-Far (PFMF), PFMN, PNMF, and Pictorial-Near-Motion-Near (PNMN). The pigeons were always reinforced in probe trials, regardless of which response icon they pecked. The pigeons completed 20 test sessions in which accuracy on training trials exceeded 80%. Between test sessions, at least one training session was conducted to prevent degradation of performance due to the addition of probe trials.

#### Analysis

For each training and test condition, the proportion of “LARGE” icon choices was fitted with a sigmoidal function as a function of target size, and the point of subjective equality (PSE) was calculated.

(1) $$P_{R}=\beta_{1}+\frac{\beta_{2}-\beta_{1}}{1+e^{\frac{-(x-\beta_{3})}{\beta_{4}}}}$$

where *P_R_* = proportion of “LARGE” icon choice and *x* = target size. Within four free parameters, *β*_3_ corresponds to PSE. Linear mixed model (LMM) was fitted to the shift of PSE between the probe and training sessions, using the “lmer” function from the R package “lme4” (Bates et al., 2015). The analysis was performed for the static and dynamic conditions, respectively. The pictorial cue was a fixed factor for the static condition, and the pictorial and motion cues were fixed factors for the dynamic condition. Subject ID was used as a random factor for both conditions. Standardized coefficients (β_std_) were calculated as effect size for each fixed effect of LMM. One-sample *t* test was conducted to analyze if PSE shifted significantly from training for each condition of static and dynamic stimuli. To test whether adding the motion parallax cue improved size constancy, amounts of illusion were calculated for static and dynamic conditions, respectively. The amount of illusion is the difference of PSE shift between PF and PN for the static condition and between PFMF and PNMN for the dynamic condition. LMM was fitted to amount of illusion with the stimulus dynamicity as a fixed and subject ID as a random factor.

### Results and Discussion

The subjects were trained between 39 and 66 sessions before advancing to test sessions; for each bird, mean accuracy on the final three training sessions was 86.7% (84.5%–89.2%). Their performances did not deteriorate following insertion of probe trials in test sessions (85.8%–90.0%).

#### Pigeons See a Corridor Illusion With Static Stimuli

**Figure 2.**
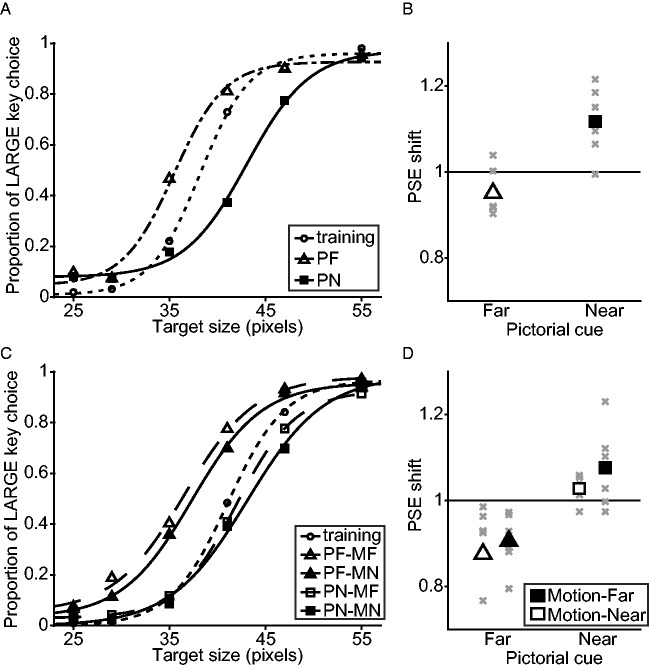
Results of Experiment 1. (A, C) Proportion of “LARGE” key choice was plotted as a function of target size for static (A) and dynamic conditions (C), respectively. Curved lines represent fitted sigmoidal functions for each condition. (B, D) PSE shift from training condition for static (B) and dynamic conditions (D). Marker positions represent mean PSE shifts across individuals, and marker colors and shapes represent conditions depicted in A and C. Gray crosses represent individual data. PF = Pictorial-Far; PN = Pictorial-Near; PFMF = Pictorial-Far-Motion-Near; PFMN = Pictorial-Far-Motion-Near; PNMF = Pictorial-Near-Motion-Far; PNMN = Pictorial-Near-Motion-Near condition; PSE = point of subjective equality.

Figure 2A and B represents the results for the static condition. Figure 2A represents the proportion of large icon choices for the static stimulus as a function of target size. Figure 2B represents the PSE shift of two test conditions from training. The pigeons overestimated the target size in the static stimulus when the pictorial cue was far and vice versa. There was a significant effect of the pictorial cue on PSE shifts, *F*(1, 5) = 63.118, *p* = .000, β_std_ = 0.794. PSE shifts in PN were significantly above 1.0, *t*(5) = 3.526, *p* = .017, *d* = 1.439. PSEs in PF condition were smaller than those in training but the difference was not significant, *t*(5) = 2.169, *p* = .082, *d* = 0.886. These perceptual biases are consistent with the corridor illusion in humans and other primates (Barbet & Fagot, 2002, 2007; Imura et al., 2008; Imura & Tomonaga, 2009), indicating that size constancy based on pictorial visual depth cues is a shared cognitive process between primates and pigeons.

#### Pictorial Cue Precedence in Pigeons

Figure 2C and D represents the results of dynamic condition. Figure 2C represents the proportion of responding on the large icon as a function of target size for training and four test conditions for dynamic stimuli. Figure 2D represents the PSE shift of four test conditions from training. The pigeons overestimated the target size in the dynamic stimulus when the pictorial cue was far and vice versa. However, the motion parallax cue had little effect on their response. There was a significant effect of the pictorial cue on PSE shifts, *F*(1, 5) = 39.591, *p* = .001, β_std_ = 0.668. Neither the effect of the motion parallax cue nor the interaction with the pictorial cue was significant—main effect, *F*(1, 5) = 2.847, *p* = .152, β_std_ = 0.130; interaction, *F*(1, 5) = 0.165, *p* = .701, β_std_ = 0.071. PSE shifts in PFMN condition were significantly below 1.0, *t*(5) = 3.504, *p* = .017, *d* = 1.430. In the remaining three conditions, PSE values did not differ significantly from training—PFMF, *t*(5) = 2.516, *p* = .053, *d* = 1.027; PNMF, *t*(5) = 2.196, *p* = .079, *d* = 0.897; PNMN, *t*(5) = 1.959, *p* = .108, *d* = 0.800. These results indicate that size constancy in pigeons is driven mainly by pictorial depth cues, while motion parallax cues have little effect, consistent with findings in humans (Luo et al., 2007; Watt & Bradshaw, 2003). Amounts of illusion in the dynamic condition (*M* = 0.200, *SD* = 0.052) were not different from those in the static condition (0.168: 0.052), *F*(1, 5) = 0.980, *p* = .368, β_std_ = 0.221, indicating that motion parallax does not contribute to size constancy in pigeons.

## Experiment 2 (Humans)

Experiment 1 demonstrated that pigeons prioritize pictorial cues over motion parallax for size constancy, as observed in humans (Luo et al., 2007; Watt & Bradshaw, 2003). In Experiment 2, humans performed a size adjustment task using the same stimuli as in Experiment 1; we needed to confirm pictorial precedence in humans using our stimuli.

### Methods

#### Subjects

Six adult humans including one of the authors (Y. H.) participated (three males, 23 to 30 years old). They had normal or corrected-to-normal vision, and five participants were naïve to the purpose of the study. They were informed about the purpose of the study and gave verbal consent in accordance with the ethical guidelines in the Declaration of Helsinki.

#### Apparatus

The experiments were controlled by a personal computer (DELL, Optiplex 980) running MATLAB with the Psychtoolbox extensions (Brainard, 1997). Stimuli were displayed on a 24-in. LCD monitor (BENQ, ET-0027-B) with 1,920 length and 1,080 height pixels resolution running at 60 Hz refresh rate. The participant’s head was not stabilized by a chin rest; participants were instructed to view the stimulus at their preferred distances.

#### Stimuli

**Figure 3.**
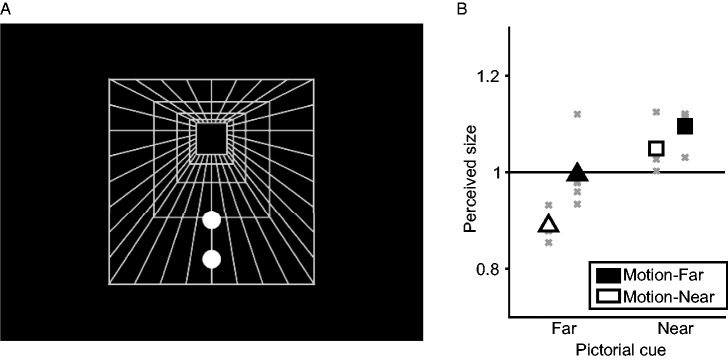
Stimulus and Results of Experiment 2. (A) The corridor illusion stimulus used in Experiment 2. A corridor background and two white circles moves horizontally to provide motion parallax cue. One circle, the comparison, was on the center of the corridor floor. The other circle, the sample, was placed on the upper or lower part of the floor. In this figure, the sample was placed on the lower part. Note that the two circles have the same size in this figure. If the corridor illusion occurs, the upper circle would be perceived to larger than the lower circle. (B) Group mean of perceived size of target circle for each condition. Gray crosses represent mean values of each individual data.

The stimulus configuration was almost the same as that of the dynamic condition in Experiment 1 (Figure 3A, see also Supplementary Movie S1), except that two white circles were simultaneously presented. One circle, the comparison, was presented at 135 pixels height from the lower edge of the corridor and moved at 70 pixels width. The size of comparison was chosen randomly from 15 to 65 pixels in diameter. The second circle, the sample, was presented above or below the comparison (±95 pixels, PF and PN conditions, respectively) and moved slower or faster than the comparison (±40 pixels width, MF and MN, respectively). The size of sample was 29, 33, 37, 43, or 49 pixels.

#### Procedure

Each trial started with the presentation of the background grid for 2 s. Then, two circles, the sample and comparison, appeared simultaneously on the monitor. One could change in size (the comparison), and the other was always the same size (the sample). The participant was instructed to adjust the size of the comparison circle (range was 15–60 pixels) to match the sample circle in image size using up and down arrow keys on a keyboard. The time to adjust was not limited. After the perceived sizes of both circles appeared identical, the participant pressed the enter key, and after 3 s, the next trial started. All participants completed 80 trials: 20 trials for each stimulus condition (2 pictorial × 2 motion parallax conditions).

#### Analysis

We calculated the perceived size as the size of the sample circle divided by the size of the adjusted comparison circle. Perceived size means were calculated for each condition individually. LMM was fitted to the perceived size means with pictorial and motion parallax cues as fixed factors and subject ID as a random factor, using the “lmer” function from the R package “lme4” (Bates et al., 2015). One-sample *t* test was conducted to analyze if perceived size differed from physical size for each condition separately. To test the species difference directly, LMM was fitted to the pooled data of PSE shifts in the pigeon experiment and perceived sizes in the human experiment, with pictorial cue, motion parallax cue, and species as fixed factors and subject ID as a random factor.

### Results and Discussion

The sample stimulus appeared larger than its actual size when the pictorial cue was far and vice versa (Figure 3B). These were significant effects of pictorial and motion parallax cues on perceived size—pictorial cue, *F*(1, 5) = 52.108, *p* = .000, β_std_ = 0.924; motion cue, *F*(1, 5) = 18.386, *p* = .008, β_std_ = 0.611. The interaction between cues was not significant—*F*(1, 5) = 2.833, *p* = .153, β_std_ = 0.299. Perceived size in PFMF was significantly below the physical size (*M* = 0.889, *SD* = 0.026), *t*(5) = 10.549, *p* = .000, *d* = 4.306. Perceived size in PFMN was not significantly different from physical size (0.995: 0.065), *t*(5) = 0.182, *p* = .863, *d* = 0.074. Perceived sizes in PNMF and PNMN were significantly above the physical sizes—PNMF, 1.049: 0.041, *t*(5) = 2.946, *p* = .032, *d* = 1.203; PNMN, 1.096: 0.033, *t*(5) = 7.038, *p* = .001, *d* = 2.873. These results suggest that size constancy in humans depends more on pictorial cues than motion parallax cues, as found in previous studies (Gregory, 1970; Luo et al., 2007; Wade & Hughes, 1999; Watt & Bradshaw, 2003).

We also tested whether the effects of pictorial and motion parallax depth cues were different between the two species. A statistical analysis with pooled data from the two experiments revealed significant effects of pictorial and motion parallax cues—pictorial cue, *F*(1, 10) = 74.578, *p* = .000, β_std_ = 0.781; motion cue, *F*(1, 10) = 15.652, *p* = .003, β_std_ = 0.517. Neither the effect of species nor the interaction with other factors was significant—species, *F*(1, 10) = 1.578, *p* = .238, β_std_ = 0.063; Species × Pictorial, *F*(1, 10) = 0.822, *p* = .386, β_std_ = 0.037; Species × Motion, *F*(1, 10) = 1.649, *p* = .228, β_std_ = 0.323; Species × Pictorial × Motion, *F*(1, 10) = 1.823, *p* = .207, β_std_ = 0.253. These findings indicate that the extent to which pictorial and motion parallax cues contribute to size constancy is not different between pigeons and humans.

## General Discussion

The first aim of the present study was to assess a corridor illusion in pigeons. In Experiment 1, we found that size constancy using pictorial depth cues occurred in pigeons, similar to humans and other primates (Barbet & Fagot, 2002, 2007; Imura et al., 2008; Imura & Tomonaga, 2009). This result suggests that the size constancy is a fundamental visual function across sighted vertebrates.

Our finding appears consistent with previous reports that pigeons see a Ponzo illusion, which is a size illusion induced by two simple converging lines (Fujita et al., 1991, 1993). Fujita et al. (1991) found little effect of perspective by adding convergent outer lines and concluded that distances between targets and inducers are important. Thus, the Ponzo illusion in pigeons is likely to work on the basis of gradient cue rather than perspective cue. In our corridor stimulus, by contrast, it is unknown which of these depth cues contributes to size constancy in pigeons. It is necessary to manipulate gradient and perspective cues separately in future studies to establish whether the Ponzo illusion and corridor illusion are based on the same mechanism.

The present study also investigated the relative effects of pictorial and motion parallax depth cues on the corridor illusion in pigeons. From Experiments 1 and 2, it is clear that both pigeons and humans depend more on pictorial depth cues than motion parallax depth cues for size constancy. When pictorial and motion parallax cues signified contradicting depth directions, both species perceived the object size on the basis of pictorial cues, consistent with human studies in which participants judged the object size (Luo et al., 2007; Watt & Bradshaw, 2003), or the three-dimensional structure of object (Gregory, 1970; Wade & Hughes, 1999).

Previous studies in pigeons and baboons demonstrate additive effects of pictorial depth cues (Barbet & Fagot, 2007; Cavoto & Cook, 2006). In contrast, adding motion parallax cue did not enhance the corridor illusion in pigeons. This suggests that various types of pictorial cue (e.g., perspective cue, gradient cue) equally contribute to 3-D reconstruction of visual information, whereas motion parallax is less used than pictorial cue at least for size constancy.

The precedence of pictorial cue over motion parallax cues might be due to a difference in reliability between these cues. Rogers and Giani (2010, p.44) suggested that pictorial cues override motion parallax cue, because

motion parallax is best thought of as providing information about the relative rotation of an object or scene with respect to the observer, and the previous empirical evidence makes it clear that the human visual system does not make any strong assumption about the stability or even rigidity of objects over time.

Thus, humans discard that assumption when motion parallax cues contradict other depth cues and perceive an illusionary motion in trick artworks (Gregory, 1970; Wade & Hughes, 1999). The same thing could be seen in our dynamic stimulus: In PFMN condition, a target appears to slide faster on a farther point of the corridor, and in PNMF condition, it appears to slide slower on a nearer point of the corridor. Our results suggest that pigeons and humans cope similarly with multiple visual depth cues based on their reliabilities to construct a veridical 3-D representation.

The precedence of pictorial over motion parallax cues in both pigeons and humans can be explained from analogous visual information processes in the two species. Size constancy in primates is processed along the ventral visual stream, using binocular or pictorial depth cues (Frassinetti et al., 1999; Tanaka & Fujita, 2015; Xia et al., 2017; for review, see Sperandio & Chouinard, 2015). Feedback signals are sent to the primary visual cortex, and receptive fields are shifted consistent with perceived object size (He et al., 2015; Murray et al., 2006; Ni et al., 2014). We speculate that motion parallax cues have little effect on size perception because they are processed in the dorsal visual stream (Kim et al., 2016). Interestingly, pigeons also process static and dynamic visual information separately, although they use phylogenetically different visual pathways than primates for visual processing (Cook et al., 2013; Nguyen et al., 2004, but see Stacho et al., 2016). The similarity in the selectivity of depth cues between the species may reflect similar ways of processing static and dynamic visual features separately.

We found a species difference in the effect of motion parallax cues on the corridor illusion: significant in humans but not in pigeons. We feel that this does not truly reflect a species difference in size constancy, for some reasons. First, when analyzing the pooled data from Experiments 1 and 2, we found no significant interaction between species and motion parallax. We acknowledge that this point is weak for the evidence of species similarity because it might be due to the small sample size (*n* = 6 for each condition), and absence of statistical significance does not mean the absence of effect itself. Second, changes in moving speed of the target were smaller in Experiment 1 (±8 pixels) than Experiment 2 (±40 pixels). The manipulation of motion parallax in the pigeon experiment might have been too small to change the perceived size. Furthermore, other differences in experimental settings could affect the behaving results (e.g., pixel sizes of monitor were different for both experiments).

As mentioned earlier, we found no significant effect of motion parallax in pigeons. However, this does not mean that pigeons are insensitive to motion parallax cue. Motion parallax cues are important for pigeons given that their lateral position of their eyes rules out use of binocular disparity cues (McFadden, 1993), and their flexible neck and head movements always produce motion parallax cues (Davies & Green, 1988). Therefore, pigeons might use motion parallax cues for something other than size constancy. Some animals use motion parallax cues for bodily motor control (Stewart et al., 2015; Wallace, 1959; for review, see Kral, 2003). Given that pigeon’s pretectal neurons in the accessory optic system, which processes visual motion caused by self-motion, are sensitive to the visual depth defined by motion parallax (Xiao & Frost, 2013), pigeons might use motion parallax for visuomotor control during flying and landing.

One limitation in our study is that motion parallax was represented by simple translational movements of external objects, not synchronized with self-movements. This leaves ambiguity about depth structure from motion parallax: The background grid appears to be a concave corridor but sometimes appears to be a convex truncated pyramid. It is known that fixational eye movements related to self-movements are important for disambiguating depth order from motion parallax (Nadler et al., 2008). We do not know how pigeons gaze the dynamic corridor and target in our study. Van der Willigen et al. (2002) demonstrated in owls transfer of depth discrimination between binocular disparity and motion parallax cues. However, owls’ performance deteriorated when motion parallax cues were not synchronized with the birds’ head movements. Future studies should investigate how pigeons use self-generated motion parallax by presenting stimuli coupled with their movements in a closed-loop manner (Stewart et al., 2015; van der Willigen et al., 2002; Wallace, 1959).

## Conclusion

This study investigated the presence of size constancy in pigeons and its characteristics in relation to depth cue selectivity. The results showed that pigeons see a corridor illusion on the basis of size constancy with pictorial depth cues. As in primates, motion parallax had little effect on size constancy in pigeons. These findings suggest that size constancy characteristics are highly similar in pigeons and primates including humans, despite the differences in their phylogeny and neuronal visual processing systems.

## Supplemental Material

IPE911408 human data - Supplemental material for Dynamic Corridor Illusion in Pigeons: Humanlike Pictorial Cue Precedence Over Motion Parallax Cue in Size Perception Click here for additional data file. Supplemental material, IPE911408 human data for Dynamic Corridor Illusion in Pigeons: Humanlike Pictorial Cue Precedence Over Motion Parallax Cue in Size Perception by Yuya Hataji, Hika Kuroshima and Kazuo Fujita in i-Perception

IPE911408 pigeon data - Supplemental material for Dynamic Corridor Illusion in Pigeons: Humanlike Pictorial Cue Precedence Over Motion Parallax Cue in Size Perception Click here for additional data file. Supplemental material, IPE911408 pigeon data for Dynamic Corridor Illusion in Pigeons: Humanlike Pictorial Cue Precedence Over Motion Parallax Cue in Size Perception by Yuya Hataji, Hika Kuroshima and Kazuo Fujita in i-Perception
